# Infection prevention in endoscopy practice: comparative evaluation of re-usable vs single-use endoscopic valves

**DOI:** 10.1016/j.infpip.2021.100123

**Published:** 2021-01-27

**Authors:** L. Pasquale, A. Maurano, G. Cengia, P. Da Massa Carrara, B. Germanà, M.G. Graziani, G. Manes, A. Pisani, M. Golia, E. Marciano, L. Rodella, L. Schiffino, C. Gandolfo, C. Terrosi, M.G. Cusi

**Affiliations:** a Gastroenterology and General Medicine Unit, Civil Hospital ‘Frangipane’ di Ariano Irpino, Avellino, Italy; b University Hospital, Mercato S. Severino, Salerno, Italy; c Digestive Endoscopy Unit, ASST Garda, Manerbio Hospital, Manerbio, Italy; d Gastroenterology and Digestive Endoscopy, Azienda USL Toscana Centro, Pistoia, Italy; e AULSS1 Dolomiti, Belluno Hospital, Belluno, Italy; f Gastroenterology and Digestive Endoscopy, S. Giovanni Addolorata Hospital, Rome, Italy; g Gastroenterology and Digestive Endoscopy, ASST Rhodense, Garbagnate Milanese and Rho Hospital, Garbagnate Milanese, Milan, Italy; h IRCCS de Bellis, Castellana Grotte, Bari, Italy; i Diagnostic and Endoscopic Surgery Unit, Monza Hospital, Monza, Italy; j Pisana University Hospital, Presidio Cisanello, Pisa, Italy; k Surgical Unit, Borgo Trento Hospital, Piazzale Stefani, Verona, Italy; l Digestive Endoscopy Unit, G.B. Grassi Hospital, Ostia, Rome, Italy; m Santa Maria alle Scotte University Hospital, Siena, Italy; n Virology Unit, Department of Medical Biotechnologies, University of Siena, Siena, Italy

**Keywords:** Endoscopy, Re-usable valves, Single-use valves, Safety, Efficacy, Infection

## Abstract

Re-usable air/water and suction valves used in endoscopes often demonstrate risk of infection. To the authors' knowledge, the safety and efficacy of re-usable and single-use valves have not been compared to date. As such, a laboratory investigation was undertaken to compare the safety and efficacy of re-usable and single-use valves at 11 Italian endoscopy sites. Safety was evaluated by analysing the rinse liquid of reprocessed re-usable valves ready for use, and efficacy was assessed based on the completion of endoscopic procedures without valve malfunction. This study found significantly lower contamination of single-use valves compared with re-usable valves (0 vs 29.1%, respectively; *P*=0.007) and similar efficacy (97.6 vs 98.8%, respectively; *P*=ns). Microbiological analysis of the rinse liquid of reprocessed re-usable valves identified various surviving micro-organisms and highlighted their potential pathogenicity. Such data suggest that sterile single-use valves may be safer than re-usable valves, and have comparable performance.

## Introduction

Re-usable valves (air/water and suction) used in endoscopes are multi-component, structurally complex devices; as such, cleaning and disinfection are extremely difficult [[1], [2], [3]]. Re-usable valves are supplied in a non-sterile state, and need to be cleaned and disinfected after each use in accordance with specific procedures recommended by the manufacturers. Cleaning and disinfection are among the most critical elements when reprocessing an endoscope and its accessories. In fact, more than 30 steps can be required for correct manual cleaning and disinfection of valves [4]. As such, the risk of infection related to these accessories should not be underestimated, and this study aimed to quantify and qualify these risks. In addition, re-usable valves are subject to risk of clogging and/or leakage due to re-use and deterioration over time.

In contrast, single-use valves are supplied in a sterile state with barcodes for traceability in order to reduce the risk of cross-contamination; moreover, as they are not re-usable, they are not subject to wear and tear over time, and efficacy is predictable and constant during an endoscopic procedure. To the authors' knowledge, the safety and efficacy of re-usable and single-use values have not been compared previously, so this investigation was conducted. Particular focus was placed on defining the level of contamination and the percentage of samples that were contaminated after reprocessing, and identifying any potential pathogens among the detected micro-organisms.

The aim of this study was to compare the safety, efficacy and cost of re-usable and single-use endoscopic valves (air/water and suction), used in accordance with current procedures at each study centre without any patient involvement. As such, it was not a clinical study.

## Methods

This prospective, multi-centre survey was conducted at 11 Italian public endoscopy units that adhered to the proposals of the Italian Society of Digestive Endoscopy and the Italian Society for Surgical Endoscopy. These societies promote compliance of endoscopy centres with national and international guidelines, including the reprocessing of endoscopes and their accessories. This study did not adopt a specific reprocessing methodology as an inclusion criterion, instead assessing the usual practice at each centre. Data on the different elements of reprocessing, including washing, disinfection and storage of endoscopic valves, were collected and analysed as independent variables. This may explain the variability in the percentage of contaminated valves between the 11 study centres; for example, Centre No. 7 used ultrasound washing, steam sterilization and an automated endoscope reprocessor, and this combination of reprocessing modalities seems to play an important role in explaining why this centre had a significantly lower percentage of contaminated valves compared with other centres, see Table II).

### Primary safety endpoint

The primary safety endpoint was measurement of microbial contamination of re-usable valves, selected at random from reprocessed valves and ready for use in endoscopic procedures. The outcome was the type of micro-organisms found and the percentage of contamination. Analysis was performed in a central laboratory where all samples were processed.

In each centre enrolled in this study, the study monitor (a biologist who had been trained by the microbiologists of the central laboratory in charge of the study) required the staff in charge of reprocessing to provide 10 pairs of re-usable valves that had been reprocessed according to the centre's standard procedures, and ready for use in endoscopic procedures.

After the valves had been collected, the study monitor rinsed the valves and collected the rinse liquid in a separate tube for each valve. Subsequently, tubes containing rinse water were shipped to the central laboratory for microbiological analysis according to a standardized procedure. For each valve, the date and time of tube shipment were recorded.

In consideration of the fact that the procedures for rinsing, shipping and analysis of valves had been standardized, but environmental differences remained between the centres (e.g. place where re-usable valves were rinsed), a pair of single-use valves (Defendo; Cantel, Minneapolis, MN, USA), just taken out of the packet, underwent the same rinsing procedure to check for any environmental contamination (negative control) and the rinsing procedure itself (16 single-use valves were checked overall).

The samples taken in the centres were analysed at the central laboratory in order to detect: coagulase-negative staphylococci (CNS), *Staphylococcus epidermidis*, *Pseudomonas* spp., *Salmonella* spp., *Shigella* spp., *Escherichia coli*, *Enterococcus* spp., *Yersinia* spp., *Klebsiella* spp., *Legionella* spp. and fungi. Other organisms were also identified and reported.

### Primary efficacy endpoint

The primary efficacy endpoint was assessed with both objective and subjective elements. Objectively, the number of valves (re-usable and single-use) that had to be replaced during an endoscopic procedure due to malfunctioning was recorded and analysed, and subjectively, the operator was asked to assess the efficacy of each valve using a Likert scale.

At each centre enrolled in this study, data regarding re-usable valves in use at the facility were recorded (both objectively and subjectively) for 4 consecutive weeks (Phase A). Subsequently, data for single-use valves were recorded (objectively and subjectively) for 4 additional consecutive weeks (Phase B).

Data recording in Phase A or B could be interrupted before the end of the 4-week period if the number of recorded procedures exceeded 50.

For each endoscopic procedure carried out over the 8-week study period, the following data were recorded: start and end times of examination; examination performed without the need for valve replacement (yes/no); type of examination (colonoscopy, gastroscopy, endoscopic retrograde cholangiopancreatography, other); result on Likert scale for valve efficacy (air/water and suction); type of valve used; number and type of valves replaced during the procedure due to malfunctioning; method of storage of valve; possibility to trace procedures undertaken previously with the same valve; valve cleaning and disinfection procedure; and time needed to reprocess the valve (re-usable valves only).

### Secondary endpoint

The secondary endpoint of this study was a comparative analysis of the purchase and reprocessing costs, and the traceability of valves.

For each centre enrolled in this study, the following data were recorded: purchase price of re-usable valves (average unit cost per pair of valves) in the 12 months preceding the start of the investigation); number of re-usable valves purchased in the 12 months preceding the start of the investigation; hourly cost of personnel involved in endoscopic practice; professional qualifications of personnel involved in endoscopic practice; and average cost of the products used for cleaning the valves.

During this investigation, each centre collected data exclusively about the use of valves and endoscopic procedures (as part of usual practice at each centre). No data regarding patients were collected.

### Specifications of microbiological analysis and procedures

Re-usable valves (already reprocessed in accordance with usual practice at each centre) were rinsed according to the following specifications. Samples were collected by the same health worker and all procedures were conducted while wearing sterile gloves. Microbiological sampling was obtained by multiple rinses of the air/water and suction valves. Ten millilitres of sterile saline buffer was used for each valve. The liquid was passed through the channels of the valve using a sterile syringe, and the fluid, flushing off the distal tip, was collected in a 50-mL tube. A sterile endoscope brush was also rubbed on each channel of the valve in order to collect any debris, and left in the tube with the collected liquid. The samples were labelled, stored at 4°C and sent to the central laboratory for microbiological testing.

The samples were analysed immediately on arrival using culture-based methods. After a mild vortex, the brush from each sample was removed and the samples were centrifuged at 4.696 *g* for 15 min at 4°C. The supernatant was recovered and stored at 4°C for possible further analyses. The pellet was resuspended in 600 μL of sterile saline buffer and inoculated equally on blood agar (bioMérieux, Marcy l’Etoile, France), MacConkey agar (bioMérieux), Sabouraud agar (bioMérieux) and Legionella agar (bioMérieux) plates. Sabouraud agar was incubated at 35° C for 5 days. Blood, MacConkey and Legionella agar were incubated at 37° C for 48 h. Plates were analysed every 24 h for rapid identification of growing bacteria. Semi-quantitative evaluation of bacterial growth classified samples as: no growth; 1–10 colony-forming units (CFU)/mL; 10–100 CFU/mL; and 100–1000 CFU/mL. Bacterial identification was undertaken using matrix-assisted laser desorption/ionization time-of-flight (MALDI-TOF) mass spectrometry (VITEK; bioMérieux). No virological analysis was undertaken.

The central laboratory analysed the samples of rinse liquid, blinded to the centre of origin or the content of the tubes (i.e. whether from re-usable or single-use valves).

### Statistical considerations

In terms of the primary safety endpoint, a sample size of 200 valves for analysis was adopted, aiming to obtain a confidence interval (CI) for the percentage of contaminated valves ≤15%.

In terms of the primary efficacy endpoint, a sample of at least 600 procedures was identified to obtain a CI for the percentage of problematic procedures ≤1.6%. Descriptive statistics were used to summarize data as frequencies (categorical variables). Fisher's exact test was used to evaluate differences between groups. Logistic regression was performed to evaluate the risk of the presence of micro-organisms with regards to the storage method. *P*<0.05 was considered to indicate statistical significance. Data analysis was performed using Stata Version 16.0 (StataCorp, Collage Station, TX, USA).

### Collected data

For the primary safety endpoint, data from 219 samples (203 samples from re-usable valves and 16 samples from single-use valves) were collected to assess methods and sampling from 11 endoscopy centres in Italy (five centres in northern Italy, three centres in central Italy and three centres in southern Italy).

For the primary efficacy endpoint, data from 1121 endoscopies (567 performed with re-usable valves and 554 performed with single-use valves) were collected. The majority of procedures were colonoscopies (51.68% with re-usable valves and 53.43% with single-use valves) and gastroscopies (41.62% with re-usable valves and 41.34% with single-use valves).

## Results

### Primary safety endpoint

In total, 29.06% of re-usable valves (Table I) showed some form of microbiological contamination, while the sterile single-use valves showed total absence of contamination.

**Table I:** Presence of micro-organisms

Micro-organisms	Re-usable valves	Single-use valves
Yes	59	0
	29.06%	0%
No	144	16
	70.94%	100%
Total	203	16

Fisher's exact test *P*=0.007.

95% confidence interval of the percentage of presence of micro-organisms in re-usable valves: 22.92%–35.83%.

Analysis of the reprocessing methods for re-usable valves (Table II) demonstrated significantly fewer micro-organisms after manual cleaning plus ultrasound and disinfection in an automated endoscope reprocessor (and sterilization in a steam autoclave), compared with manual cleaning and disinfection in an automated endoscope reprocessor (5.6% vs 35.6%, respectively; *P*=0.009). No reprocessing methodology, however, was able to guarantee 100% safety in the re-usable valves.

**Table II:** Presence of micro-organisms by cleaning/disinfection method

Presence of micro-organisms	Yes	No	Total	95% CI	
Manual cleaning + automated endoscope reprocessor	58	105	163	From	To
Row %	35.58%	64.42%	100%	28.2	43.4
Column %	98.31%	86.07%	90.06%		
Manual cleaning with ultrasound + automated endoscope reprocessor + steam autoclave	1	17	18		
Row %	5.56%	94.44%	100%	0.14	27.29
Column %	1.69%	13.93%	9.94%		
Total	59	122	181%		
Row %	32.6%	67.4%	100%		

CI, confidence interval.

Fisher's exact test *P*=0.009.

With regards to the storage method used for re-usable valves, no significant difference in contamination was found for the different methods considered (simple cabinet 36.7% vs ventilated cabinet 20%; *P*>0.001). Finally, with regards to brushing or not brushing, no significant difference in contamination was observed (32.3% vs 13.7%, respectively; *P*=0.3). The logistic model suggested that storage in a simple cabinet increased the risk of the presence of microorganisms 2.8-fold compared with storage in a ventilated cabinet, although this was not significant (*P*=0.07).

Micro-organisms detected with frequency exceeding 5% on re-usable valves were Gram-negative bacteria (23.1%), CNS (13.25%) and *Micrococcus luteus* (9.62%). In 13.3% of cases (11/83), these micro-organisms were considered to be dangerous pathogens for humans, particularly in immunocompromised individuals.

Besides microbial agents, debris (probably of organic origin), presumably as a result of inadequate cleaning, was found. Failure to remove foreign material from the inside and outside of a device can interfere with the effectiveness of subsequent disinfection and/or sterilization. Organic matter provides a breeding ground for the growth of bacteria, and provides protection from the lethal effects of cleaning, disinfection and sterilization. Failure to loosen and remove bioburden from an endoscope facilitates the formation and growth of biofilms and may be a risk factor for the control of infection [5]. Twenty-two different organisms – bacteria and fungi – were detected (Table III). The majority were common skin commensals, CNS, *Micrococci* spp., environmental micro-organisms, non-pathogenic Gram-negative bacteria and fungi. However, potential human pathogens were also detected such as *Rothia mucilaginosa* (10 CFU/mL), a Gram-positive coccus, that – although present in the normal microbiota of the human mouth and upper respiratory tract – is recognized as an opportunistic pathogen that mainly affects immunocompromised hosts with severe pneumonia [6]. Among the identified staphylococci, *Staphylococcus lugdunensis* (10 CFU/mL) and *S. epidermidis* (10–1000 CFU/mL) – normal inhabitants of human skin and mucous membranes – have long been dismissed as culture contaminants because of their potentially important role as pathogens (Table III). They can infect immunocompromised patients with medical devices by creating a biofilm and causing bacteraemia and sepsis [7,8].

**Table III:** Micro-organisms in endoscopic valves

	CFU/mL								
Species	Center	1	2	3	4	7	8	9	10
*Actinomyces viscosus*		10							
*Aspergillum flavus*								10	
*Bacillus simplex*			10						
*Corynebacterium afermentas*			10						
*Escherichia coli*			10						
*Kitococcus sedentarius*								10	
*Kocuria kristinae (Rothia kristinae)*					10				
*Microbacterium aoyamense*						10			
*Micrococcus luteus*			10		10		10	10	10
*Paracoccus yeei*			10		10				
*Proteus mirabilis*			10						
*Roseomonas mucosa*			10						
*Rothia dentocariosa*		10							
*Rothia mucilaginosa*			10						
*Staphylococcus capitis*		10	100	10	10				
*Staphylococcus epidermidis*			1000	10	1000			10	10
*Staphylococcus hominis*				10					10
*Staphylococcus lugdunensis*		10						10	
*Staphylococcus pasteuri*		10			100				
*Staphylococcus warneri*					10				
*Stenotrophomonas maltophilia*							10		
*Trichophyton rubrum*					10				
Environmental fungi								10	
Environmental Gram-negative bacteria			100					10	

CFU, colony-forming units.

Among the Gram-negative bacteria, only *Stenotrophomonas maltophilia* (10 CFU/mL) was identified as a human pathogen. *S. maltophilia* is an emerging environmental Gram-negative multi-drug-resistant organism that is most commonly associated with respiratory infections in humans, but can also cause other serious infections such as meningitis, endocarditis, urinary tract infection and osteomyelitis [9]. With regards to fungi, *Aspergillum flavus* (10 CFU/mL), second only to *Aspergillus fumigatus* as a cause of human invasive aspergillosis [10], was detected in a set of endoscopic valves.

Regarding typical bacteria found in lower gastrointestinal endoscopy, some colonies of *E. coli* and *Proteus* spp. were found in a few samples. In addition, several small colonies identified as Gram-negative bacteria were not identified by MALDI-TOF mass spectrometry.

### Primary efficacy endpoint

Data collected during 1121 endoscopic procedures did not show a significant difference between the efficacy of re-usable valves (98.8%) and single-use valves (97.6%) in terms of completion of the endoscopic procedure without the need for valve replacement (Table IV).

**Table IV:** Overall success of endoscopy (i.e. procedure completed without problems caused by valve malfunction)

	Re-usable valves	Single-use valves	Total
Yes	559	538	1.097
	98.76%	97.64%	98.21%
No	7	13	20
	1.24%	2.36%	1.79%
Total	566	551	1.117

Fisher's exact test *P*=0.180.

Note: four not available.

Analysis of the rating scale (Likert scale) for air/water valves showed a difference in the distribution of satisfaction between re-usable and single-use valves, with greater variability in satisfaction/dissatisfaction for re-usable valves (89% and 86% ‘totally agreed’ with the statement ‘The valve worked well’ for re-usable and single-use valves, respectively, while 9% and 13% ‘partially agreed’ for re-usable and single-use valves, respectively; *P*=0.039). On the contrary, for suction valves, no significant difference in the distribution of satisfaction/dissatisfaction was found between re-usable and single-use valves (*P*=0.2).

### Secondary endpoint

Regarding the consumption of resources, information was collected regarding the cost of products used for reprocessing, hourly cost of staff, and purchase price of re-usable valves. The average cost of a pair of re-usable valves, from data provided by five centres, was 185 € [standard deviation (SD) 46.5 €, median 116 €]. Reprocessing costs showed greater variability; the average cost, from data provided by 10 centres, was 26 € (SD 34 €, median 9.2 €).

Data collected showed extreme variability between centres, so significant analysis was not possible. With regards to traceability, the data showed that, in all cases, it was not possible to directly trace endoscopic examinations performed previously with a particular re-usable valve. The only possible way to maintain the traceability of re-usable valves would involve keeping valves with the same endoscope throughout the course of their life cycle.

## Discussion

Despite attention paid to the reprocessing of endoscopic instruments, results of previous studies have suggested that can be difficult to clean the valves efficiently due to their complex structure. In this study, microbiological tests performed on re-usable valves (reprocessed according to the usual procedures at the participating centres) showed the presence of potentially pathogenic micro-organisms after reprocessing. The source of micro-organisms could be due to the failure to remove or inactivate contamination resulting from use, or contamination introduced after decontamination. It was not possible to confirm the origin of the contamination. Staff training and standardization of the procedure are often insufficient to guarantee complete disinfection of all parts of an endoscope. It should be acknowledged that the current practice for endoscope reprocessing is disinfection rather than sterilization. The poor traceability of some phases of the process also makes it difficult to trace specific problems. This hinders processes to implement decisive improvement and corrective actions (e.g. patient recall in the case of cross-contamination).

Analysis of the impact of consumption and resource use showed high variability for re-usable values (mainly due to the wide range of costs reported by the study centres). Despite this variability, the data collected suggested that costs associated with reprocessing re-usable valves are not negligible, significantly affecting the total cost.

In conclusion, this study found that despite following stringent reprocessing guidelines, contamination of re-usable values is possible, while single-use valves are sterile and may provide a higher degree of patient safety.

The reprocessing of endoscopic instruments – a complex and poorly traced procedure – includes manual phases and is therefore at risk of operator variability. Single-use valves enable simplification of this procedure and a reduced risk of contamination. No significant differences in operator satisfaction and procedural effectiveness were found between re-usable and single-use valves. Single-use valves may therefore represent a safe and effective alternative for patients in routine practice. More precise cost evaluation is desirable as this would enable a cost/effectiveness comparison between the two types of valve.
